# Evidence for multiple introductions of an invasive wild bee species currently under rapid range expansion in Europe

**DOI:** 10.1186/s12862-020-01729-x

**Published:** 2021-02-05

**Authors:** Julia Lanner, Fabian Gstöttenmayer, Manuel Curto, Benoît Geslin, Katharina Huchler, Michael C. Orr, Bärbel Pachinger, Claudio Sedivy, Harald Meimberg

**Affiliations:** 1 Institute for Integrative Nature Conservation Research, University of Natural Resources and Life Sciences Vienna (BOKU), Gregor-Mendel-Straße 33, 1180 Vienna, Austria; 2 Insect Pest Control Laboratory, Joint FAO/IAEA Division of Nuclear Techniques in Food & Agriculture, Wagramer Straße 5, 1400 Vienna, Austria; 3 MARE Marine and Environmental Sciences Centre, Faculdade de Ciências, Universidade de Lisboa, Camop Grande, 1749-016 Lisboa, Portugal; 4 IMBE, Aix Marseille Université, Avignon Université, CNRS, Marseille, France; 5 Key Laboratory of Zoological Systematics and Evolution, Institute of Zoology, Chinese Academy of Sciences, 1 Beichen West Road, Beijing, 100101 China; 6 Heinrichstrasse 267A, 8005 Zurich, Switzerland

**Keywords:** *Megachile sculpturalis*, Haplodiploidy, Genotyping-by-amplicon sequencing, Multiple introductions, Transportation vectors

## Abstract

**Background:**

Invasive species are increasingly driving biodiversity decline, and knowledge of colonization dynamics, including both drivers and dispersal modes, are important to prevent future invasions. The bee species *Megachile sculpturalis* (Hymenoptera: Megachilidae), native to East-Asia, was first recognized in Southeast-France in 2008, and has since spread throughout much of Europe. The spread is very fast, and colonization may result from multiple fronts.

**Result:**

To track the history of this invasion, codominant markers were genotyped using Illumina sequencing and the invasion history and degree of connectivity between populations across the European invasion axis were investigated. Distinctive genetic clusters were detected with east–west differentiations in Middle-Europe.

**Conclusion:**

We hypothesize that the observed cluster formation resulted from multiple, independent introductions of the species to the European continent. This study draws a first picture of an early invasion stage of this wild bee and forms a foundation for further investigations, including studies of the species in their native Asian range and in the invaded range in North America.

## Background

Invasion occurs when a species is accidentally or intentionally introduced to and establishes within such a new environment. But invasive pollinators, like invasive bee species, often deviate from the negative perspective of invaders, especially when it comes to pollinators of crops. Bees are generally considered beneficial for the pollination services they provide, but they may have surprising negative impacts on local ecosystems where they do not naturally occur [1–4]. Negative impacts can take different forms, including through disrupting plant-pollinator networks, competition with native species for nest sites or other resources, pollinating invasive plants, the introduction of novel pathogens, and economic losses [5–8]. Among bees, those which nest in cavities opportunistically are disproportionately invasive, i.e. *Afranthidium (Immanthidium) repetitum*, and, relatedly, wood-nesting bees make up larger proportions of the fauna in more remote islands due to higher dispersal abilities [9, 10]. In these ways, invasive bees can be promising study organisms for investigating range expansion and dispersal patterns [11].Given increasing reports of invasions that come with globalization of human society [12], it is crucial that we learn more about the dynamics of bee invasions to better protect native and managed ecosystems.

**Fig. 1:**
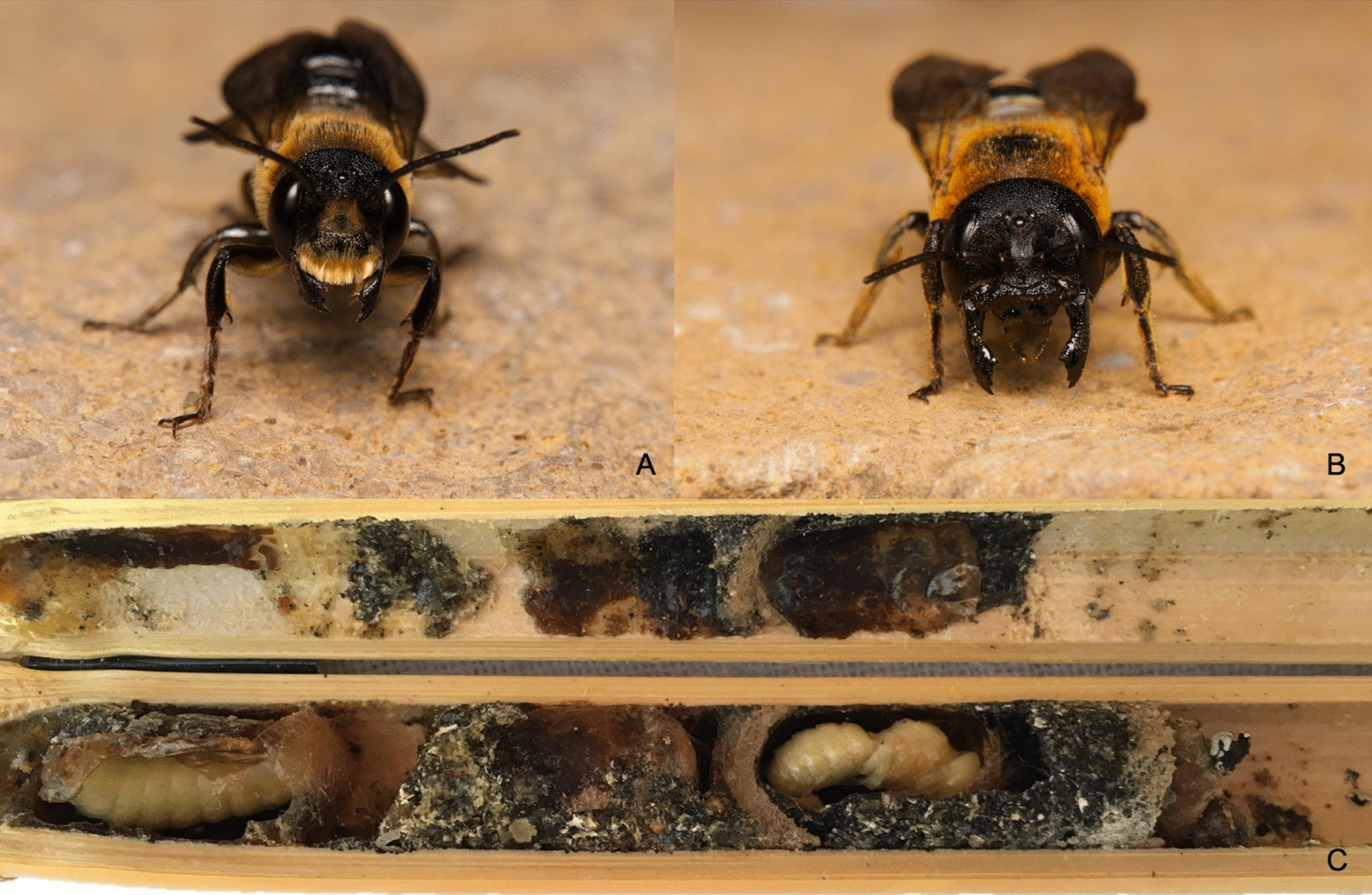
Sex dimorphism of *Megachile sculpturalis*: **a** males have a three-toothed mandible and shiny yellow hair on their supraclypeal plate, and lack a metasomal scopa. **b** females are bigger, with a largely black metasomal scopa except for the first two sternites where hairs are yellow. **c** As a protandric species, male larvae are positioned at the front in the nests and emerge earlier than females

*Megachile* (*Callomegachile) sculpturalis* Smith, 1853, one of hundreds of species amongst *Megachile* [13], is the first translocated bee species to become established in Europe. *Megachile sculpturalis* is a protandric wild bee with an active season from June to mid-September in Europe [14–17]. Males body size ranges between 12 and 22 mm, and females between 20 and 28 mm. Both sexes are characterized by their narrow body, bright orange hairy thorax hair and dusky wings (Fig. 1) [18, 19]. *Megachile sculpturalis* is a cavity-nesting wild bee using preexisting holes in wood. The species naturally occurs in East-Asia (Japan, China, South-Korea and Taiwan), but was first established outside its native range in the eastern USA around 1994, and is now considered as invasive species [14, 20, 21]. Although it is classified as a pollen generalist, this polylectic species presents a strong preference for pollen of *Styphnolobium japonicum* and *Ligustrum* sp. in Europe. Both plants are native to Asia and often used as ornamental plants in human habitats [16, 17, 22]. The bee species shows aggressive behavior towards the local bee fauna from time to time, and events of direct evictions have been observed in the US and in Europe [23, 24]. A recent study detected a negative correlation between its presences in trap nests and the emergence of native bee species raising the question of the magnitude of the impact of *M. sculpturalis* on the local bee populations [25].

In Europe, this species was first recognized in a small city near Marseille (Allauch, France) in 2008 [26]. There are relatively few examples of other invasive aculeate species in Europe, besides *Vespa velutina* first recognized in 2005 also in South-France, and *Megachile disjunctiformis* found in northern Italy in 2011 [27–29]. *Megachile sculpturalis* most likely found its way to Europe as a stowaway on ships while nesting in dead wood [26, 30]. Following its initial discovery in France, the species was reported from northern Italy in 2009 [16]. In the following years, *M. sculpturalis* colonized many European countries remarkably fast. Its range expansion from France and Italy continued to Spain, Switzerland, Germany, Austria, Hungary, Slovenia, Croatia, Serbia, Bosnia Herzegovina reaching now to the Crimean Peninsula [16, 22, 23, 30–37]. A recent compilation of records from our collections and a citizen science approach, show range extensions to eastern locations, including Vienna (2017) [38], Gyöngyös (2015) [33], and Belgrade (2017) [32]. This suggests that the current distribution pattern in Europe was attained by two distinctive mechanisms of dispersal. First, after its introduction in South-France, *M. sculpturalis* probably colonized most countries by stepwise dispersal. Additionally, accidental transport in wood could explain the large distances-over hundreds of kilometers-between isolated reports eastward [23].

The invasion of *M. sculpturalis* had been tracked relatively well, because of the conspicuous size of the species (for an European bee) and its typical association with anthropogenic habitats [23]. Thus, we are able to investigate the early spread of this species in real-time and generate baseline genetic diversity data to better understand the structure and colonization pathways, before widespread admixture within the invasive range occurs. On the basis of genetic diversity, the success of translocated populations might be linked not only to the number of founding individuals but also the number of different colonization events [39–41]. Population genetic analyses are therefore an important tool to study the patterns and intensity of dispersal over geographic ranges [42–45].

To conduct such analyses, allele frequencies from selected neutral marker loci are compared to assess gene flow, which is a crucial parameter studying population dynamics and invasion biology [39, 46, 47]. Great advances have been made in the study of genetic diversity in non-model organisms over the last decade. Codominant datasets from microsatellites or simple sequence repeats (SSRs) were the standard for assessing connectivity and genetic structure between populations for a long time [45, 48, 49]. The main advantage of the genotyping by amplicons sequencing (SSR-GBAS) approach is the direct accessibility of the sequence of an allele without the need to indirectly use size as a proxy. Here, we call the method SSR-GBAS, as in earlier studies SSR-GBS was used which led to confusion because of the frequent view of using GBS exclusively for RAD methods [50, 51]. Variants using similar allele call methods have also been termed SSR-Seq [52]. The single-base-pair resolution enables enhanced sensitivity in detecting alleles and hidden size-homoplasy with a greater potential for individual identification as well as the ability to resolve low genetic diversity and spatial patterns at very fine scales [53–55]. SSR-GBAS can also be applied for species with large genomes, where little or no genetic data are available. Another major advantage of parallel sequencing is the high number of samples that can be included in single sequencing runs, therefore circumventing some additional shortcomings of microsatellites aside from their exploring only length-polymorphisms [50, 56, 57]. The approach is especially promising for samples providing little or degraded DNA [55], which is often the case when working with small organisms such as wild bees. Overall, this type of marker is ideal for exploring the invasion dynamics of exotic species.

Here, we present and validate a set of SSR loci as markers suitable for a genotyping by amplicon sequencing approach. We use these markers for a first investigation of genetic variations of *M. sculpturalis* across Europe. The degree of connectivity between widespread populations is explored to detect signs of one or multiple colonizing events in Central Europe. The present study sets the baseline for further population genetic analyses, including specimens not only from Europe but also from its native range in Asia and North America. It should serve as a model for a deeper understanding of the interdependencies of successful colonization dynamics and mechanisms of dispersals, such that we may better predict and control the spread of invasive species in the future.

## Results

### Output statistics

To study the genetic variability of European *M. sculpturalis* populations, 48 SSR markers were designed and tested on 81 samples of European *M. sculpturalis* specimens. Out of all 48 markers, two (FB12 and FB47) were excluded due to an unusually high number of alleles as a possible consequence of duplication or scoring errors. The marker set included 20 pentanucleotide (two were excluded) and another 28 tetranucleotide motifs. Primer information is summarized in Table 1, including polymorphism information content, number of different alleles and unbiased, expected heterozygosity (uHe).

**Table 1:** The marker set for *Megachile sculpturalis* including primer sequence (F = forward and R = reverse), repetition motif, four primer mixes (PM1—4), amplification length (ASR), polymorphic information content (PIC) and unbiased expected Heterozygosity (uH_e_L) per locus

Locus	Primer sequence (5′–3′)	Repetition motif	Primer mix	ASR	PIC	Number of alleles	uH_e_L
FB1	F: TTTTTCGTCGTCACGCTG	CCTTT-9	PM 1	412–422	0.487	3	0.564
	R: CGGAGTTAAAGGAAGGGAAA						
FB2	F: ACCGACGCTATTAAAATTGTC	AGAAG-19	PM 3	407–437	0.558	3	0.638
	R: TCCACGAAAGAGTTCTTTGT						
FB3	F: GTGAAATCGACGCGAAAC	TTTCG-10	PM 4	421–441	0.501	3	0.592
	R: ATTGGTCTTCTGGTTCGTTT						
FB4	F: GTAGACACCTCGTGACTTTT	GAGAG-9	PM 3	412–422	0.278	3	0.312
	R: ATAAGCCGCGACACAAATT						
FB5	F: CTGTCGAGTCGGACCTTT	TCTTT-12	PM 1	442–447	0.322	2	0.406
	R: ATCGACGAGTTTTTGTACGA						
FB6	F: ACGTTCTTCTTACGATTCTTC	ATAGA-8	PM 3	463–478	0.704	5	0.754
	R: GGAATGCCTTTTCGAGTTTC						
FB7	F: AAGAGAACAATAAGTCGAACG	TCTTT-7	PM 4	360–400	0.566	4	0.643
	R: TGAACGTACGTAATTTGCAT						
FB8	F: TCGTACGAGAAACGAAAGG	TTTCG-10	PM 3	390–410	0.492	3	0.585
	R: AGTCAGACGAGTAGTTAGGC						
FB9	F: ACCCCACGAATGTTAACG	CGACG-8	PM 1	398–433	0.519	5	0.573
	R: TTTACGGCCGAGTTTTCC						
FB10*	F: CGTCAAAGGCTACCGTATAA	AACAG-14	PM 3	436–441	0.2	2	0.227
	R: CCTTCAATGTTCAAGGTGAA						
FB11	F: CCACTTTATCCGTTTGTTCG	AATCG-9	PM 3	415–430	0.603	4	0.658
	R: ACGGCGAAATCGAGTCTTA						
FB13	F: TTGAATCTCTGCTCTGTGAC	TTTTC-11	PM 3	442–467	0.576	3	0.656
	R: GCGGTGATATTAGACTCGTA						
FB14	F: GTCGAGCGATTGGAGTAATC	TACTC-8	PM 1	420–495	0.509	4	0.554
	R: CGTACCTCGAAAATACGCT						
FB15	F: GAACAGAAGGTTTGTTCGC	TCGTA-9	PM 4	440–485	0.43	5	0.477
	R: TTCCATCTGGCACGATAG						
FB16	F: CTGAAGTCATTCGGGTAAGA	CGAAA-9	PM 3	412–427	0.478	4	0.538
	R: TCGTACTTTTCACCCTGAAA						
FB17	F: CGGGCATGAACGAATATTCT	TTCGT-7	PM 1	420–455	0.491	4	0.543
	R: TACTTTTAACGTCGCGTATT						
FB18*	F: GAACGTTGACCAAGTGGATT	AAAGA-8	PM 3	400–400	0.229	2	0.266
	R: AGTTCGAGCTGTCACTTTTT						
FB19	F: CTAATTGCCATCGAGCCAG	CGTTC-8	PM 3	445–470	0.501	5	0.557
	R: GACGATCTTGGTTAAAACAGT						
FB20*	F: CTTCTCGTTCGAGGATCATT	CTTC-8	PM 1	435–435	0	1	0
	R: GGTCAAGGTAAAGGAAGGAG						
FB21	F: AAGCATCGTACCTCGGTATA	ACGA-5_GAAC-11	PM 1	378–410	0.678	5	0.73
	R: GTGTTCCCTTTAAAACTCGC						
FB22	F: CTCTGTCTCAGGTTAGTGTG	TCTT-13	PM 3	412–424	0.294	5	0.311
	R: AATAGAGCGCATTACCGATT						
FB23	F: GATATCGATCCCATCCGAAA	GTGC-12	PM 3	384–400	0.735	7	0.774
	R: TCTCACGATGATTATACGTCC						
FB24	F: CAAACTTTCCTGGTACCGG	TTTC-11	PM 4	390–402	0.538	3	0.618
	R: ACGATTAAATCATTTCGGTTGA						
FB25	F: TTGAAATTCAACGTATGCGC	AGAA-11	PM 1	434–434	0.556	5	0.635
	R: GAACGGCGAAACTTCTATTC						
FB26	F: AATCGAAAAAGAAACACGGG	GAAG-7	PM 3	429–433	0.581	3	0.661
	R: AGAAACTCACCTTGTCCATC						
FB27	F: ATTGTTGAGGCGAATAACCT	ATGT-10	PM 4	403–443	0.677	5	0.728
	R: TCGTTAAGAATGAATGAACGA						
FB28	F: CCTTGGTCTCGTCGTTATTA	TCTT-8	PM 3	416–422	0.699	5	0.749
	R: GTCGTTTAACCGAGACGC						
FB29	F: TACCTTTCGTCAAAGATGCA	TATT-8	PM 3	432–444	0.425	4	0.536
	R: ATAGAAGCATGTCAACACCC						
FB30	F: CGGGTACGACAAGGATTAAA	CCAA-9	PM 4	427–435	0.592	3	0.671
	R: ATCAGCCTAGAGTGTAGAGG						
FB31	F: GATCACCTACGTAATGCTGT	GAAC-7	PM 1	421–421	0.535	4	0.611
	R: CGCCACTCGCATAAAATTAA						
FB32	F: TCCCGGGCAAAGATAAATAT	GTTC-11_TTCG-5	PM 3	409–441	0.676	5	0.728
	R: ATATATCGCCTCGTGTTACG						
FB33	F: GAAGAGCCTATAGACCCTGT	CCTT-10	PM 3	400–420	0.621	5	0.692
	R: AACTATTGCCGAGTTATCCG						
FB34*	F: TTTCATAATTTACGCGTCTCTC	ATCT-8	PM 1	400–400	0	1	0
	R: ATATCGAATCTACCTCTGTGC						
FB35	F: TTAAACTCCATTACGCGTCA	ATAG-12	PM 3	445–453	0.407	3	0.493
	R: CATCGCGGACAATAAACTTT						
FB36	F: AACTGATCCCCTGCCATT	GCGT-9	PM 4	399–415	0.66	4	0.717
	R: TGTTCTAGCGCATCTGATTC						
FB37	F: AGCAAAAACGTGAAGAATGA	GCGT-12	PM 3	373–401	0.58	3	0.659
	R: AAATTTTCGACCACATACGC						
FB38	F: ACCTTAGTCGTTAGTAGCCT	TCTG-7	PM 3	417–421	0.537	3	0.621
	R: CGGAAGGAAATTTCGTACGA						
FB39	F: GCTGACTTGCACACAAATTT	AAGG-10_AAAG-5	PM 3	406–426	0.66	5	0.715
	R: CGATCCCGTTATCTTCGATT						
FB40	F: GCAAGAAACAAAAACCGTTG	CTCA-18	PM 1	385–421	0.522	4	0.6
	R: CGTTAGTCTGACAGTTTTCG						
FB41	F: TTTGCTACAAAACACTGGAC	AGAA-7	PM 3	430–450	0.716	5	0.763
	R: GTACCTCTCGAACTTCCTTT						
FB42	F: TTGTGTCACCTTTAATCGGT	TTGC-8	PM 4	444–508	0.563	6	0.604
	R: GGATAAAAATCCGTGCGAAA						
FB43	F: TGTTCGCCTCTAGATCGATA	TCCT-11	PM 3	419–443	0.624	5	0.69
	R: GCGTTAATCAAATGGTTCGA						
FB44	F: ACGCGAATAATTTACGATGAC	CGTT-10	PM 3	399–407	0.522	4	0.572
	R: GATTAAACAAAAGCGGCAAC						
FB45	F: GAATCGCCAAAAGTGCATAA	GAAG-14	PM 1	438–454	0.376	3	0.491
	R: TACTTTCGAGTCTACTTGCC						
FB46	F: CCGGGGGAAAGTTCAATTTA	GAAA-9_AGAA-11	PM 4	435–507	0.525	6	0.593
	R: TTGTAATTTGCAATCGCGAT						
FB48	F: GAAAAGGAAAGTCGAAACGG	CGTT-9	PM 4	373–405	0.557	4	0.634
	R: TACCAGATAGAAACGCGATG						

### Genetic diversity of European populations

The partial Illumina MiSeq run resulted in 3,043,426 paired reads for the library analyzed with an average of around 41,000 reads per sample.

Of the 81 total specimens, seven samples were missing ten or more markers and were excluded from further analysis. In total 74 samples were included. The sample group FR represented the highest number of polymorphic loci with 95.65%, followed by the sample group CH 93.48% and SFR with 89.13% and finally, VIE with 67.39%, which is probably a result of the uneven sample sizes across regions. Specimens from Vienna had the highest number of private alleles (VIE = 0.383), in contrast to low levels in samples from the group CH = 0.191 and SFR = 0.021. The remaining sample group FR had 0.298. This pattern is also reflected by the level of genetic distance between the groups (GD, Table 3). Population diversity statistics were first applied including all 74 samples. Due to the haplo-diploid system, females are heterozygous, and males are supposed to be homozygote, which should decrease H_o_. When females (n = 42) were separated from the males, we saw a similar picture as for all samples: the Ho was lower than He in all groups and for females overall, but not for the group VIE (Table 2).

**Table 2:** Summary statistics for sample groups (Switzerland = CH; France = France; SFR = South-France; VIE = Vienna) including all (_T_) samples and only female specimens (♀): number of samples (N); number of alleles (Na); effective number of alleles (Ne); observed (H_o_), expected (H_e_) and unbiased expected heterozygosity (uH_e_)

Group		NT	N♀	NaT	Na♀	NeT	Ne♀	HoT	Ho♀	HeT	He♀	uHeT	uHe♀
CH	Mean	21.717	11.804	2.804	2.783	2.133	2.092	0.148	0.272	0.478	0.469	0.489	0.490
	SE	0.157	0.080	0.127	0.124	0.094	0.093	0.014	0.026	0.029	0.028	0.029	0.029
FR	Mean	35.196	19.587	3.326	3.283	2.132	2.127	0.074	0.104	0.483	0.482	0.490	0.494
	SE	0.328	0.180	0.146	0.138	0.09	0.091	0.006	0.009	0.027	0.027	0.027	0.028
SFR	Mean	6.891	4.891	2.119	2.565	2.119	2.175	0.134	0.188	0.471	0.476	0.508	0.530
	SE	0.055	0.056	0.098	0.123	0.098	0.109	0.017	0.024	0.029	0.031	0.032	0.034
VIE	Mean	8.826	4.957	1.456	1.761	1.456	1.413	0.282	0.321	0.251	0.242	0.266	0.269
	SE	0.072	0.030	0.069	0.094	0.069	0.056	0.037	0.041	0.031	0.029	0.033	0.032

Genetic differentiation (Pairwise F-statistics = Fst) was low between samples from the group SFR, FR and CH, in comparison to values between samples from VIE. Multiple approaches generated similar patterns of this differentiation, e.g. GD and sHua between populations (Table 3).

**Table 3:** Pairwise F statistics (Fst), number of effective migrants (Nm) and genetic distance (GD), mean Shannon Values over Loci (sHua) values for the four sample groups: South-France (SFR), France (FR), Switzerland (CH) and Vienna (VIE) incorporating 74 samples

Group 1	Group 2	Fst	Nm	GD	sHua
CH	FR	0.037	6.489	87.905	0.064
CH	SFR	0.044	5.370	84.097	0.051
FR	SFR	0.071	3.296	94.571	0.063
CH	VIE	0.340	0.486	121.293	0.512
FR	VIE	0.329	0.510	121.497	0.369
SFR	VIE	0.363	0.439	125.238	0.624

**Fig. 2:**
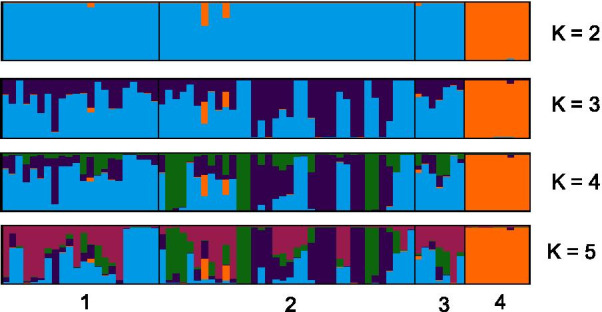
Principal coordinates analyses from the four sample groups. For the above panel individuals are colored according to the sample groups: Switzerland (CH), France (FR), South France (SFR) and Vienna (VIE). The panel below based on cluster assignment probability calculations in Structure for K = 4. A sample was assigned to a certain cluster if the Q score was above 50%, otherwise was considered to be admixed

**Fig. 3:**
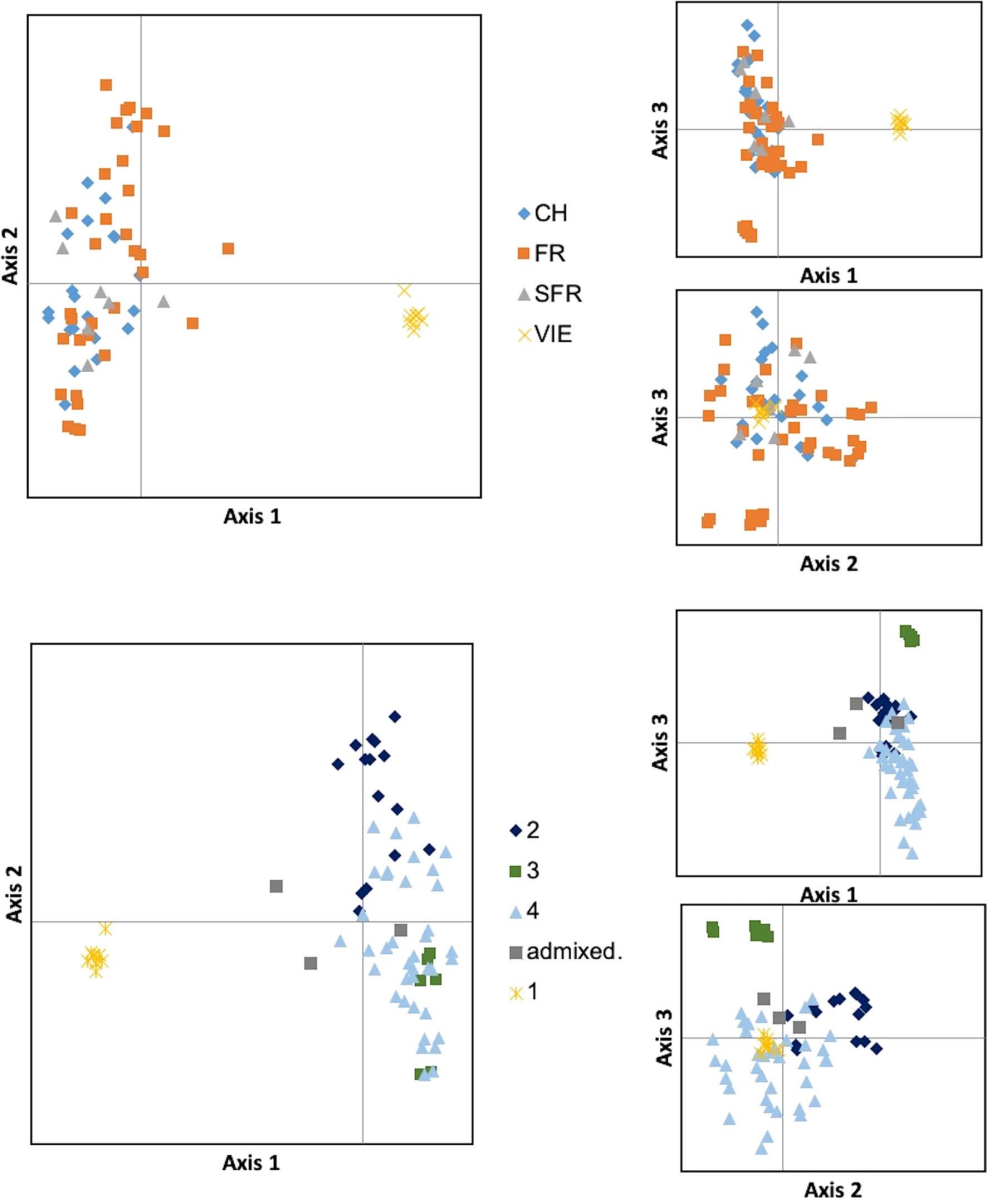
Genetic structure bar plots for *Megachile sculpturalis* based on cluster assignment probability calculations in Structure for the best K values K = 2 and K = 5 (1 = Switzerland, 2 = France, 3 = South-France and 4 = Vienna)

PCoA provided a visualization of the genetic distance patterns between individuals of the four sample groups. Samples from the groups SFR, FR and CH formed one discrete cluster. Samples from Vienna were separated from the remaining samples by relatively high distances (Fig. 2). No genetic structure was obvious in PCoA between the three sample groups SFR, FR and CH (n = 65), excluding the samples assigned to Vienna. Structure and Structure Harvester found that Delta K is highest for a K value of two, meaning that the likelihood of two different big genetic clusters is the highest (Fig. 2). The second highest likelihood is for K = 5. Generally, structure analyses were consistent with the outputs of PCoA, indicating two distinctive groups, one for the VIE samples and one for the rest, at K = 2 (Fig. 3). A higher K output indicates different clusters, most diverse in the group FR (cluster 2) followed by CH, and SFR. When cluster assignment was used as a group criterion in the PCoA, these samples grouped to some extend together. This was most clearly found for a group of six individuals from the group FR.

Overall, pairwise FST values between populations were high between Vienna and the other groups (0.33–0.36), suggesting different populations, and low between the groups from France and Switzerland (0.04–0.07; Table 3).

In an AMOVA, 21% of variation was explained by differences between geographical groups and 59% within groups and 20% within individuals. When grouping was done according to the structure analysis, 37% of the variation was described between clusters, 45% within clusters and 18% within individuals. The latter values were derived from K = 4 because of the lower number of admixed individuals. Despite the genetic structure apparent in Structure analysis and PCoA, the sign tests for bottlenecks were significant for all regions with He > Heq as well as the one-tailed Wilcoxon tests (Table 4).

**Table 4:** Sample groups Switzerland (CH), France (FR), South-France (SFR, as putative point of origin) and Vienna (VIE) were tested for signs of a bottleneck by carrying out sign tests and two-tailed Wilcoxon tests. Observed expected heterozygosity (H_e_) under HWI exceeded expected heterozygosity under mutation-drift equilibrium (H_eq_). Tests were all significant as values were below 0.01

Group	Loci with H excess		Sign test	Two-tailed Wilcoxon-test
	H_e_	H_eq_		
CH	39	21.91	0.0000	0.0000
FR	36	23.13	0.0001	0.0000
SFR	36	21.37	0.0000	0.0000
VIE	23	15.81	0.0072	0.0062

## Discussion

Besides the general threats of invasive species (i.e. translocated pathogens, overcompetition), introduced bees have the potential to impact the indigenous biodiversity by altering pollination networks. Invasive pollinators can have effects on the native flora by modifying the pollen transfer patterns, which affects the seed set of indigenous plants [58]. In addition, introduced plant species lacking a suitable pollinator, can develop from so-called ‘sleeper weeds’ to expanding populations once a suitable pollinator is introduced [59]. There are already 80 bee species around the world that occur in non-native areas. The genus *Megachile* is most frequently represented in this group [7]. Therefore, understanding introduction events, colonization pathways and promoting factors is crucial in the effort to conserve biodiversity.

Species introduction events begin at their origins, where efficient vectors for single or multiple colonization events are required. Humans most often act as vectors and transport species via commerce and trading networks [60, 61]. With ongoing globalisation, the frequency of species introductions has increased from year to year on a global scale [12]. Human-mediated dispersal is influenced by the likelihood and frequency for a certain species to infiltrate human transport and arrive alive and able to reproduce, which depends on several factors: population size, nesting behavior, biotic interactions and phenology [62–66]. In the case of *M. sculpturalis*, only long-distance transportation can explain its invasion [26, 67]. As an example, one specimen was found previously on an airplane in Hawaii arriving from Japan, although it never established there [14]. More likely, *M. sculpturalis* found its way to North America and Europe on ships following maritime trading routes [26, 30]. As the species is a cavity-nesting bee, it is assumed that wood packaging material provided shelter for overwintering larvae during transportation, like for most insects translocated between continents [65, 68]. After arrival, the species was unintentionally released in its new environment, with establishment then dependent on the balance of available resources vs. the species’ requirements. Besides long-distance introdcutions to their new environment, some invaders experience range expansions over large longitudinal distances. This stepwise dispersal pattern are in most cases man-made promoting invasion success rates [10, 69]. Humans act as ecological filters by translocating species around the world, accidentally or intentionally, whereby the likelihood of human-vectored dispersal increases with human movement and contact zones, e.g. transport networks for roads and ships, trading points and urban areas [66, 70, 71].

This theoretical framework is reflected in the location where *M. sculpturalis* was first observed in Europe. Marseille constitutes an important maritime trading point in southern France. Ships for maritime trading were also most likely the vector delivering the species unintentionally to European islands, e.g. the most recent finding on Mallorca (Balearic island, Spain; ) [72]. Similarly, *M. disjunctiformis* was found near an international trading hub (Interporto) near Bologna, Italy [29]. This second invasive wild bee recognized in Europe presents very similar ecological requirements to *M. sculpturalis* in sharing the same natural biogeography, East-Asia, and it is even a member of the same subgenus (*Callomegachile*). This supports the assumption that invaders are not random samples of the global species pool [7, 66, 73, 74]. Marker development of the founder specimen of *M. disjunctiformis* based on the method described is currently in progress.

Introduced species often require a demographic lag phase immediately following the initial colonization event, prior to full establishment and subsequent range expansion [4]. Although *M. sculpturalis* was already recognized in 2008 [26], for many years the species was reported by single or very few observations restricted to certain areas. During this lag phase, it can be challenging to gather a sufficient sample size to develop and test microsatellites to investigate their genetic structure.

By implementing SSR-GBAS, fine-scale ecological questions can be answered [45], e.g. the small-scale genetic differences found within samples from France. Further, low genetic variation for the sample group of SRF, its putative point of origin, was observed according to the unbiased expected heterozygosity. Invaders typically show low genetic variation, as only fractions of the original gene pool were translocated within the founder specimen(s). Reduced heterozygosity and effective size of the gene pool result in genetic drift fostered by inter-mating of founding individuals [75–77]. Nevertheless, organisms with haplo-diploid systems generally have the ability to overcome extreme bottlenecks. Within several hymenopterans, haploid unfertilised eggs develop to males and diploid fertilised eggs mostly into females. In the honey bee sex is determined by the *csd locus* that is heterozygous in females and homozygous in males in what is described as single-locus complementary sex determination [78]. In small groups, likewise founder populations, as a result of the decreased overall heterozygosity, diploid males are more common since diploid eggs are more likely to be homozygote for this locus and develop as males. Diploid male production was found to produce less viable and sterile males driving the population towards the edge of extinction [79, 80]. However, studies have shown that these systems have the ability to overcome the genetic load by a balanced selection on the *csd* locus, which acts as a coping strategy circumventing the extinction vortex [80, 81]. Besides a smaller effective population size and heterozygosity levels, males are under strong purging selection of exposed deleterious alleles. Therefore, even a small number of founder individuals in haplo-diploid organisms like *M. sculpturalis* can successfully invade new areas [74, 77, 82–84]. Regardless, three out of four males sampled in Vienna in 2018 were found to be diploid (Additional file 1: Fig. S1) indicating a high percentage of diploid males in the species at least in expanding or founding populations. During future investigations more specimens morphologically assigned as males will be included to investigate this context.

A polyandrous mating system is another possible explanation for the observed invasion success despite severe founder effects. Even moderate polyandry, with more than two mates, can maintain genetic diversity and enable populations to overcome founder effects [85, 86]. But in contrast to many social hymenopterans, polyandry in solitary bees is reportedly scarce, with few exceptions (e.g. Megachilidae: *Anthidium manicatum)* [87], and a female was seen to mate singly, but only once (personal observation, Lanner 2020).

The striking differences detected between Vienna and all other regions represents our most unexpected finding. The genetic structure of both females and all samples, as well as the principal coordinate analysis, differentiated these two groups clearly: a western sample group (South-France, France and Switzerland) and a second, eastern group from Vienna. Vienna contains the lowest number of effective alleles and polymorphic loci. The observed heterozygosity of the sample group VIE exceeded expected heterozygosity. However, specimens from Vienna also had the highest number of private alleles. Given these findings, it seems highly unlikely that the population in Vienna spread from the 2008 origin in France. Instead, we hypothesize that they were transported independently, meaning that Europe was invaded multiple times by this species. This would explain both the discordance between eastern and western genotypes and the remarkably fast and wide-spread distribution of *M. sculpturalis* in Europe [23]. In addition, specimens from the remaining sample groups show genetic structure indicated by the assignment likelihood using Structure, the higher between group variation in the AMOVA (when groups are defined not geographically but according to the inferred clusters) and by the respective comparisons of pairwise distances as shown in the PCoA. The origin of these distinct genetic clusters from one colonization event is highly unlikely [88–90]. Although we could not assign all individuals to a location, this shows that, besides the split between a western lineage and an eastern lineage (represented by Vienna), additional lineages might have been independently introduced. Considering the recent report of the closely related *M. disjunctiformis* in Italy, the potential of frequent recurrent introduction of this species should be high. This might be one factor for its high invasive potential [23].

Multiple introductions can facilitate successful establishment via an increased number of individuals, a broader spatial range and increased genetic diversity [40, 41, 91, 92]. Thereby, with an increasing number of introductions and individuals, the chance of establishment and range expansion also increases. With multiple sources, the established population should also be more diverse, buffering the negative impacts of genetic drift [76, 93]. Moreover, genetically diverse invasive species benefit from higher adaptive capacity, and can react more flexibly when facing challenges in their new environment [41, 94]. In some cases, with sufficient independent introductions, genetic diversity in the invaded range may be even higher than in its indigenous range [40].

### Conclusion

Microsatellite loci enabled the first insights on the demographic dynamics of this introduced wild bee. By better quantifying population genetic structuring, indirect measurements of gene flow revealed striking differences between populations within Europe. As the SSR-GBAS was applied in a relatively early invasion stage before the expected admixture between European populations took place, we were able to find tremendous genetic patterns. This east–west differentiation as well as fine-scale genetic patterns are best explained by multiple colonization events in Middle Europe. Such multiple colonization events can partly explain its invasion success in Europe. Further, it is a possible explanation for its adaptation potential to its new environment. To confidently determine the number of colonization events, future studies with larger sampling from all invaded regions of Europe will be necessary. By implementing genetic landscape modelling, we are planning to estimate the probability of migration between geographic separated populations and to test the degree of connectivity more explicitly. Furthermore, specimens from its native origin and North America will be included to produce a synthetic view of its global invasion patterns and potential. By including North American samples for genetic structure analyses, we will be able to test if European individuals are bridgehead populations, those from a previously-invaded region rather than the native range, as are often found in other hymenopterans [61]. If the primary exotic habitat contains substantial genetic diversity, it may act as a particularly potent source for another, second translocation [40, 49, 73, 95], making it crucial that we quickly better our understanding of this species ‘ invasive dynamics.

## Methods

### Sample collection

**Fig. 4:**
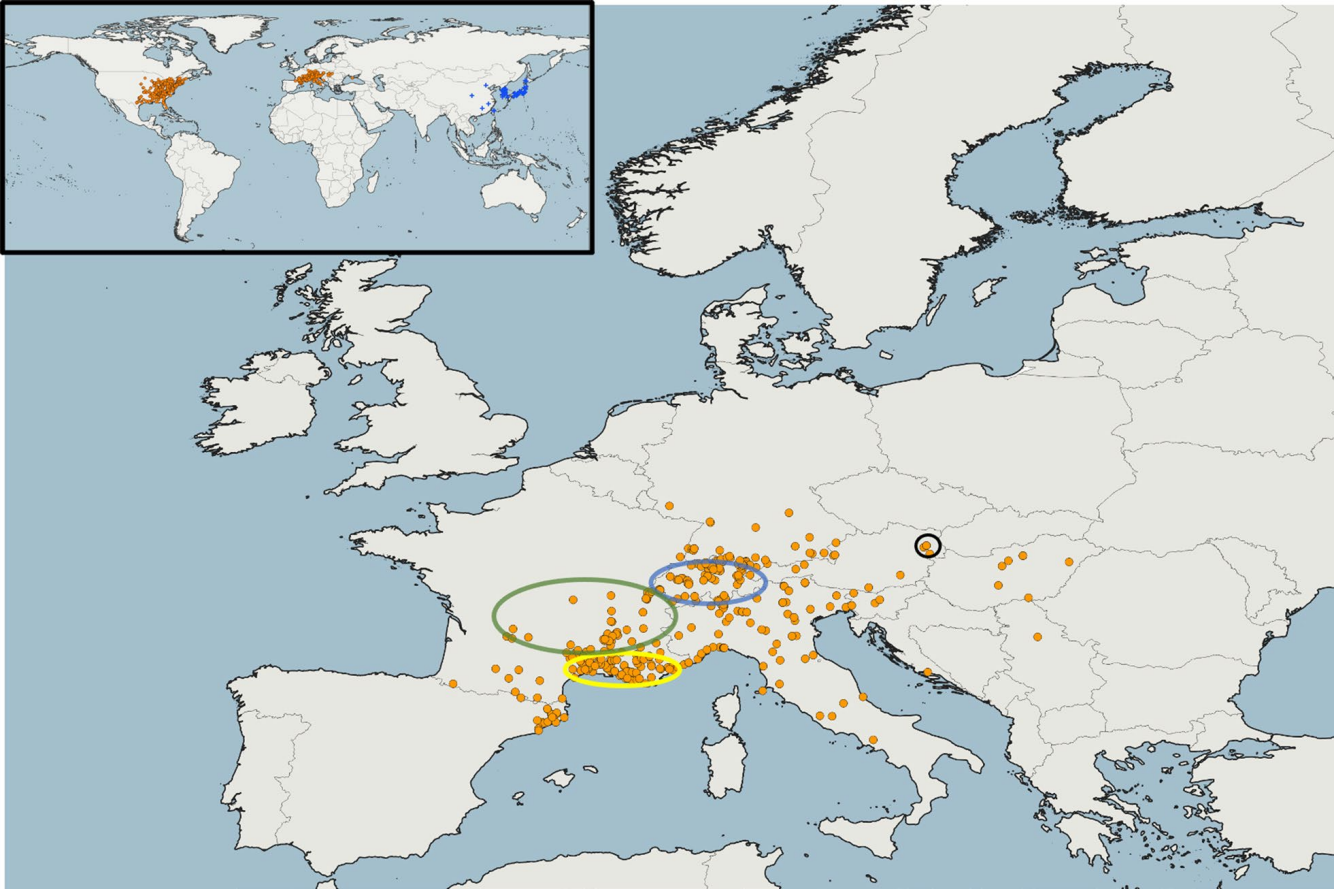
Reported European occurrences of *Megachile sculpturalis* until 2018. Records were derived from literature and verified citizen science programs (iNaturalist ©, info fauna CSCF, observation.org, GBIF©, naturgucker.de, insecte.org), including indicated sample groups from South-France (yellow circle), France (green circle), Switzerland (blue circle) and Vienna (brown circle). The map was created with QGIS for the present study

Specimens for the present study were gathered from commercially-circulated trap nests (in France and Switzerland) and an international initiated citizen science project aiming to investigate its distribution in Switzerland, Liechtenstein and Austria [23]. Samples provided by citizen scientists were collected on private properties. In  Fig. 4, all published localities are indicated until 2018 [16, 22, 23, 26, 30–37, 96–103] as well as localities mentioned in public nature platforms and communication tools related to nature topics (i.e. iNaturalist, observations.org, naturgucker.de). For tests of genetic variation, samples were organized into four sample groups reflecting geographic origins, which were used as populations in subsequent analyses (South-France = SFR, France = FR, Switzerland = CH, Vienna = VIE; Fig. 4). Specimens from trap nests are representatives from localities close to their point of first recognition (SFR, n = 7), other parts in France (n = 40), Switzerland (n = 22) and Vienna (n = 11). Coordinates from vouchers were available from 28 specimens. Samples were included as adults (n = 20) and larvae (n = 61) collected from trap nests in the study, whereas 42 adults and larvae were assigned as females and 39 individuals were assigned as males (Additional file 2: Table S1). Genetic tests were performed for all samples and due to the haplo-diploid system of hymenopterans, we segregated female larvae by their size and position within the nests from all others (as a protandrous solitary bee, females emerge later than males and were positioned at the back of the nests).

### Marker design and Illumina amplicon sequencing

Marker development was conducted according to Tibihika [51] and Curto et al. [50]. We prepared a whole DNA library (approx. insert length 400–500 bp according to Nextera XT Library Prep., Illumina, USA) and used it for a low coverage run on the Miseq platform, targeting to produce about 300,000 to 1,000,000 paired reads. Whole DNA Library construction and sequencing was performed by Service (LMU Sequencing center) based on a specimen from the founder population in South-France.

In total, 83 samples were available for DNA isolation. To extract DNA from larval samples, approximately 1 mm of tissue was used. Tissue samples from adult bees were taken by cutting off the third leg. Bee legs were ground with 5 Zirkonium beads (Zirkonoxide beads, Type ZY-P, 2.7–3.3 mm, Sigmund Lindner, Germany) per tube in a mill at maximum force (700 rpm) for 20 min. Isolation was done using the DNA tissue Kit by Macherey–Nagel GmbH & Co KG, Germany with according to the manufacturers protocol, with few modifications as follows: In each tube, 180 μL Pre-lysis buffer T1 and 10 μL Proteinase K (10 mg/mL) was added to the ground tissue, vortexed, and incubated at 56 °C overnight on a Mixing Block MB-102 (Bioer, China). Next, 10 μL RNAse (10 mg/mL) was added and the mixture incubated at 37 °C for 10 min, and subsequently at 70 °C for 10 min with an additional 180 μL Lysis buffer B3. After lysis, the samples were centrifuged with for one minute each at 1000, 2000, 4000, 8000 rpm in a Eppendorf 5430 R centrifuge (Eppendorf, Germany). The last step was carried out at 11,000 rpm for 7 min. After centrifugation, 360 μL supernatant was mixed with 180 μL Ethanol abs. and loaded onto an EconoSpinTM columns with silica membranes (Epoch Life Science, USA) and centrifuged at 8000 rpm for 1 min. The membrane was washed with 600 μL of 80% Ethanol at 12,000 rpm for 1 min and dried afterwards via centrifugation for 2 min and at 12,000 rpm after discarding the flow through. DNA was eluted at 12,000 rpm for 1 min with 50 μL with 10 Tris (pH of 8) preheated to 65 °C and incubated at least 3 min. The resulting DNA solution was used as the template for amplification.

DNA amplification was carried out by a two-step PCR approach with four sets of primer mixes (Table 1), which were multiplexed for PCR following the procedure described in Curto et al. 50. During the first PCR, the target region is amplified using specific primer pairs. The primer pairs contain additional bases at the 5′-ends. In the second PCR reaction the adapters bind to their complementary oligonucleotides in the primer pair of the first PCR reaction [104–106]. Five μL Master mix (Qiagen Multiplex PCR Kit), 1 μL primer mix with a concentration of 1 μM (forward and reverse combined), 3 μL H_2_O and 1 μL DNA were mixed. PCR run with the following program: 95 °C for fifteen minutes, 95 °C denaturation temperature for 30 s, annealing at 55 °C for one minute and elongation at 72 °C for one minute, repeating for 30 cycles. For the second PCR, 1.5 μL from each PCR product from the four multiplex reactions were pooled per sample and cleaned by mixing it with 4.3 μL AMPure XP magnetic beads (Beckman Coulter Life Sciences, USA). Washing steps as well as the second PCR elongating the PCR strands with unique indexes for sequencing, pooling the amplicons and purification followed Curto et al. 50. The libraries were sequenced in one Illumina MiSeq run targeting around 3,000,000 paired reads of 300 bp, corresponding to an average depth of 800 per sample per marker.

### Data analyses

Data analysis was done using scripts described previously and available at github.com/ mcurto/SSR‐GBS‐pipeline [51]. Sequences were processed by merging the paired reads, quality control used PEAR [107], identification of the primer and sorting the sequences by loci. Allele calls according to length and SNPs, as well as determination of heterozygote genotypes from the consensus sequences and stutter control followed the approach of Curto et al. 50. Manual control of the raw data, which is possible with these scripts, was deemed unnecessary. The pipeline results were transformed into a co-dominant matrix in GenAlex [108] format, from which it can be transformed to other population genetic software. Expected heterozygosity (H_e_), observed heterozygosity (H_o_), and number of alleles (Na), Mean Shannon Values over loci (sHua) using Log Base = 2, number of migrants, AMOVA and G-statistics were calculated with GenAlex. We also calculated Joest’s estimate of differentiation with GenAlex, as for highly polymorphic loci the Fst value depends on the allele number per locus. Pairwise Fst [109] was tested with 9999 permutations. Allelic richness was tested with the program Hp-rare [110]. Demographic changes such as population expansion or bottlenecks were evaluated using the program Bottleneck [111]. Genetic structure was tested using Structure [112] and principal coordinate analysis based on pairwise genetic distances (PCoA) between individuals as implemented in GenAlex. Structure ran for 50.000 generations of burnin followed by 100,000 generations for K values ranging from 1 to 9 with 10 replicates each. The optimal K value was accessed using the DeltaK method as implemented in Structure Harvester [113]. For a second PCoA, clusters were assigned according to the Structure results for the most frequent lineages and with a likelihood > 50%.

Finally, we tested for genetic bottlenecks with the hypothesis of bottlenecked populations showing a higher than expected heterozygosity based on Hardy–Weinberg Equilibrium (HWI) than under the mutation-drift equilibrium (H_e_ > H_eq_) [114, 115]. Number of loci with H_e_ > H_eq_ were counted and tested if regions showed signs of an increase in heterozygosity above zero by calculating sign tests and carried out one-tailed Wilcoxon tests, as in Neophytou et al. (2019). Tests were carried out in the program BOTTLENECK under the infinite allele model (IAM) [116].

## Supplementary information

**Additional file 1: Fig. S1.** Box plot of the proportion of heterozygous loci found in females from the sample groups CH (Switzerland), SRF (South-France), VIE (Vienna) and males collected in Vienna (VIE male1–4), whereas VIE males 2–4 were found to be diploid in several loci.

**Additional file 1: Table S1.** Detailed information of the samples included in the present study. Adult specimens were assigned as males = m, or females = f. As protandric species, females emerge later than males and are positioned at the cavity end: L1 = first larvae positioned at the cavity back, L2 = second larvae positioned nest to L1; Ln = larvae were counted and labeled according to their nest position.

## Data Availability

The datasets generated and/or analysed during the current study are available in the NCBI repository with the following BioProject number: PRJNA680990 and submission number SUB8642671.

## Abbreviations

SSR-GBAS: Short sequence repeat–genotyping by amplicon sequencing
PCR: Polymerase chain reaction
SRF: Sample group South-France
FR: Sample group France
CH: Sample group Switzerland
VIE: Sample group Vienna
uH_e_L: Unbiased expected heterozygosity
Na: Number of alleles
He: Expected heterozygosity
Ho: Observed heterozygosity
PCoA: Principal coordinates analysis
N: Number of samples
Ne: Effective number of alleles
uH_e_: Unbiased expected heterozygosity
H_eq_: Expected heterozygosity under mutation-drift equilibrium
Fst: Pairwise F statistics
Nm: Number of effective migrants
GD: Genetic distance
sHua: Mean Shannon Values over loci
