# Correction to: Evidence for multiple introductions of an invasive wild bee species currently under rapid range expansion in Europe

**DOI:** 10.1186/s12862-021-01765-1

**Published:** 2021-02-17

**Authors:** Julia Lanner, Fabian Gstöttenmayer, Manuel Curto, Benoît Geslin, Katharina Huchler, Michael C. Orr, Bärbel Pachinger, Claudio Sedivy, Harald Meimberg

**Affiliations:** 1 Institute for Integrative Nature Conservation Research, University of Natural Resources and Life Sciences Vienna (BOKU), Gregor‑Mendel‑Straße 33, 1180 Vienna, Austria; 2 Insect Pest Control Laboratory, Joint FAO, IAEA Division of Nuclear Techniques in Food & Agriculture, Wagramer Straße 5, 1400 Vienna, Austria; 3 Faculdade de Ciências, MARE Marine and Environmental Sciences Centre, Universidade de Lisboa, Camop Grande, 1749‑016 Lisboa, Portugal; 4 IMBE, Aix Marseille Université, Avignon Université, CNRS, Marseille, France; 5 Key Laboratory of Zoological Systematics and Evolution, Institute of Zoology, Chinese Academy of Sciences, 1 Beichen West Road, Beijing, 100101 China; 6 Heinrichstrasse 267A, 8005 Zurich, Switzerland

## Correction to: BMC Ecol Evo (2021) 21:17 https://doi.org/10.1186/s12862-020-01729-x

Following publication of the original article [1], we were notified that the legends for Fig. 2 and Fig. 3 were swapped.

The original article has been corrected.
