# Supplementary figures and images for: Evidence for multiple introductions of an invasive wild bee species currently under rapid range expansion in Europe
Source: BMC Ecol Evol. 2021 Feb 5;21:17. doi: 10.1186/s12862-020-01729-x (PMC7866639; doi:10.1186/s12862-020-01729-x)

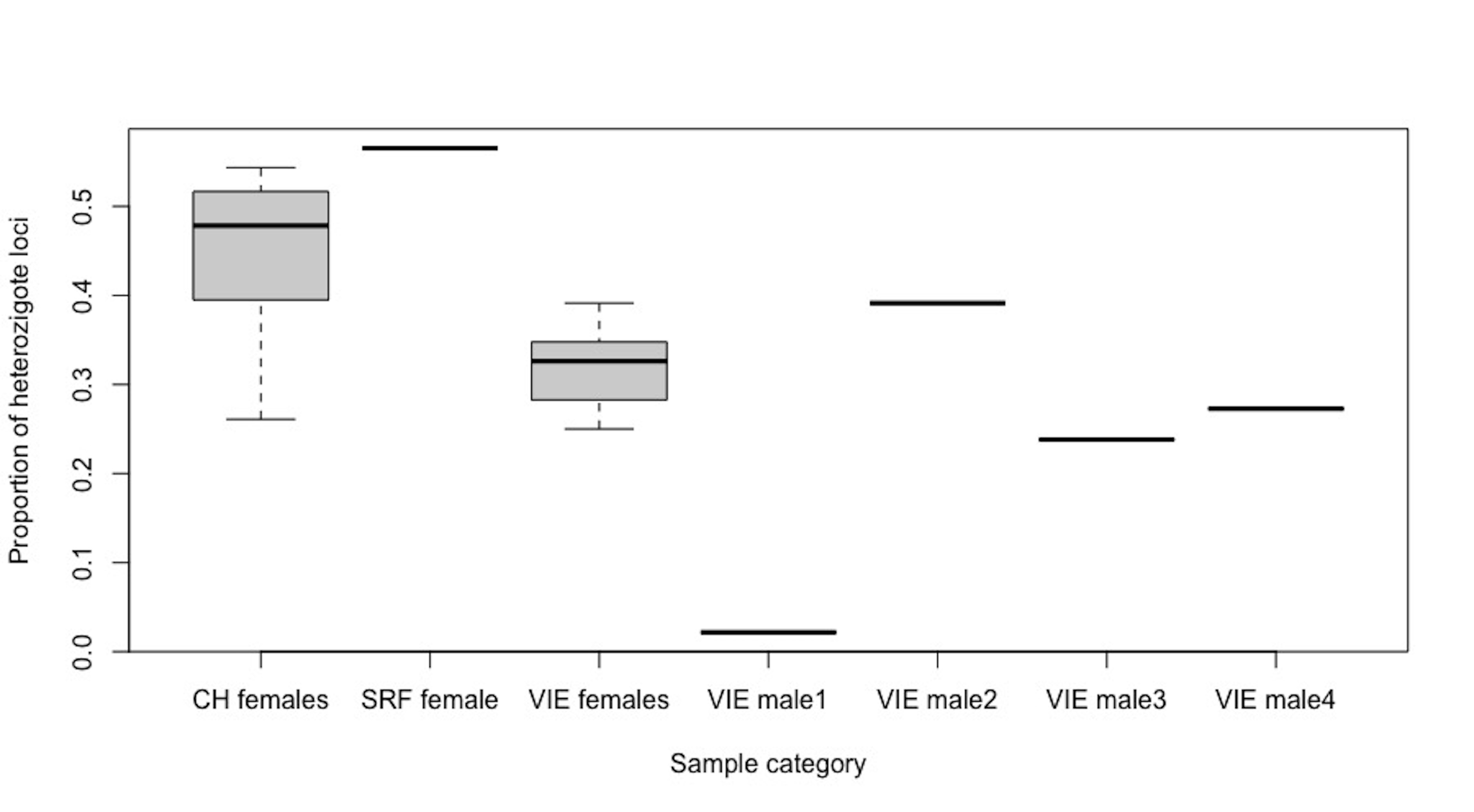

Supplement: Supplementary file 1 — Additional file 1: Fig. S1. Box plot of the proportion of heterozygous loci found in females from the sample groups CH (Switzerland), SRF (South-France), VIE (Vienna) and males collected in Vienna (VIE male1–4), whereas VIE males 2–4 were found to be diploid in several loci. [file 12862_2020_1729_MOESM1_ESM.jpeg]
