# Supplementary material for: Evidence for multiple introductions of an invasive wild bee species currently under rapid range expansion in Europe
Source: BMC Ecol Evol. 2021 Feb 5;21:17. doi: 10.1186/s12862-020-01729-x (PMC7866639; doi:10.1186/s12862-020-01729-x)
Supplement: Supplementary file 2 — Additional file 1: Table S1. Detailed information of the samples included in the present study. Adult specimens were assigned as males = m, or females = f. As protandric species, females emerge later than males and are positioned at the cavity end: L1 = first larvae positioned at the cavity back, L2 = second larvae positioned nest to L1; Ln = larvae were counted and labeled according to their nest position. [file 12862_2020_1729_MOESM2_ESM.pdf]

sample ID	sample group	year	Adult/Larvae	male/female/larvae position	Location	cooridnates N	coordinates E
CH05aL	CH	2018	L	L1	Sargans	47.048264	9.4402
CH10aL	CH	2018	L	L1	Sierre	46.292252	7.532319
MSAA-2018	CH	2018	A	f	Affoltern am Albis	47.279225	8.455325
MSF-2018	CH	2018	A	f	Frauenfeld	47.554114	8.912028
MSF2-2018	CH	2018	A	f	Frauenfeld	47.554114	8.912028
MSOaT	CH	2018	A	f	Oberhofen am Thunersee	46.730836	7.673244
MSPL-2018	CH	2018	A	f	Petit-Lancy	46.194136	6.117028
MSSaR-2018	CH	2018	A	f	Stein am Rhein	47.655819	8.857956
MSV-2016	CH	2016	A	f	Vernagaz	46.137617	7.040828
CH01bL	CH	2018	L	L2	Zurich	47.372394	8.542333
CH02aL	CH	2018	L	L1	Zurich	47.361573	8.529741
CH06aL	CH	2018	L	L1	Berne	46.95293	7.397099
CH07cL	CH	2018	L	L3	Berne	46.95293	7.397099
CH08aL	CH	2018	L	L1	Berne	46.95293	7.397099
CH08bL	CH	2018	L	L2	Berne	46.95293	7.397099
CH09aL	CH	2018	L	L1	Berne	46.95293	7.397099
CH13aL	CH	2018	L	L1	Switzerland		
CH13bL	CH	2018	L	L2	Switzerland		
CH13cL	CH	2018	L	L3	Switzerland		
CH13dL	CH	2018	L	L4	Switzerland		
CH13eL	CH	2018	L	L5	Switzerland		
CH13fL	CH	2018	L	L6	Switzerland		
MSAR-4	VIE	2018	A	f	Vienna	48.153767	16.396103
MSAR1	VIE	2018	A	m	Vienna	48.153767	16.396103
MSAR2	VIE	2018	A	f	Vienna	48.153767	16.396103
MSAR3-1	VIE	2018	A	m	Vienna	48.153767	16.396103
MSAR3-2	VIE	2018	A	f	Vienna	48.153767	16.396103
MSAR3-3	VIE	2018	A	f	Vienna	48.153767	16.396103
MSAR5	VIE	2018	A	f	Vienna	48.153767	16.396103
MSAR7-2	VIE	2018	A	m	Vienna	48.153767	16.396103
MSAR7-3	VIE	2018	A	m	Vienna	48.153767	16.396103
MSAR6	VIE	2018	A	m	Vienna	48.153767	16.396103
MSAR7-1	VIE	2018	A	m	Vienna	48.153767	16.396103
Marseille-bee	SFR	2009	A	f	Marseille	43.305	5.3972
SF01aL	SFR	2018	L	L1	South-France		
SF02aL	SFR	2018	L	L1	South-France		

SF02bL	SFR	2018	L	L2	South-France
SF03aL	SFR	2018	L	L1	South-France
SF03aL-2	SFR	2018	L	L	South-France
SF05aL	SFR	2018	L	L1	South-France
FR01aL	FR	2018	L	L1	France
FR02aL	FR	2018	L	L1	France
FR02bL	FR	2018	L	L2	France
FR02cL	FR	2018	L	L3	France
FR03aL	FR	2018	L	L1	France
FR03bL	FR	2018	L	L2	France
FR04aL	FR	2018	L	L1	France
FR04bL	FR	2018	L	L2	France
FR05aL	FR	2018	L	L1	France
FR06aL	FR	2018	L	L1	France
FR09bL	FR	2018	L	L2	France
FR10aL	FR	2018	L	L1	France
FR10bL	FR	2018	L	L2	France
FR12aL	FR	2018	L	L1	France
FR13aL	FR	2018	L	L1	France
FR13bL	FR	2018	L	L2	France
FR14aL	FR	2018	L	L1	France
FR15aL	FR	2018	L	L1	France
FR15bL	FR	2018	L	L2	France
FR17aL	FR	2018	L	L1	France
FR18aL	FR	2018	L	L1	France
FR19aL	FR	2018	L	L1	France
FR19bL	FR	2018	L	L2	France
FR19cL	FR	2018	L	L3	France
FR21aL	FR	2018	L	L1	France
FR21aL-2	FR	2018	L	L1	France
FR21bL	FR	2018	L	L2	France
FR23aL	FR	2018	L	L1	France
FR23bL	FR	2018	L	L2	France
FR24bL	FR	2018	L	L2	France
FR24cL	FR	2018	L	L3	France
FR25aL	FR	2018	L	L1	France
FR25bL	FR	2018	L	L2	France

FR26aL-2	FR	2018	L	L1	France
FR26bL	FR	2018	L	L2	France
FR20bL	FR	2018	L	L2	France
FR20dL	FR	2018	L	L4	France
FR23cL	FR	2018	L	L3	France
FR24aL	FR	2018	L	L1	France
FR01bL	FR	2018	L	L2	France
